# Testing the myth: tolerant dogs and aggressive wolves

**DOI:** 10.1098/rspb.2015.0220

**Published:** 2015-05-22

**Authors:** Friederike Range, Caroline Ritter, Zsófia Virányi

**Affiliations:** 1 Comparative Cognition, Messerli Research Institute, University of Veterinary Medicine, Vienna, Medical University of Vienna, University of Vienna, Veterinaerplatz 1, 1210 Vienna, Austria; 2 Wolf Science Centre, Dörfles 48, 2115 Ernstbrunn, Austria

**Keywords:** agonistic behaviour, aggression, tolerance, dominance, domestication

## Abstract

Cooperation is thought to be highly dependent on tolerance. For example, it has been suggested that dog–human cooperation has been enabled by selecting dogs for increased tolerance and reduced aggression during the course of domestication (‘emotional reactivity hypothesis’). However, based on observations of social interactions among members of captive packs, a few dog–wolf comparisons found contradictory results. In this study, we compared intraspecies aggression and tolerance of dogs and wolves raised and kept under identical conditions by investigating their agonistic behaviours and cofeeding during pair-wise food competition tests, a situation that has been directly linked to cooperation. We found that in wolves, dominant and subordinate members of the dyads monopolized the food and showed agonistic behaviours to a similar extent, whereas in dogs these behaviours were privileges of the high-ranking individuals. The fact that subordinate dogs rarely challenged their higher-ranking partners suggests a steeper dominance hierarchy in dogs than in wolves. Finally, wolves as well as dogs showed only rare and weak aggression towards each other. Therefore, we suggest that wolves are sufficiently tolerant to enable wolf–wolf cooperation, which in turn might have been the basis for the evolution of dog–human cooperation (canine cooperation hypothesis).

## 1. Introduction

Cooperation within as well as across species has been suggested to correlate with high tolerance and low aggression towards group members [1,2], independently of cognitive abilities [3,4]. Similarly, in domestic dogs cooperation with humans is thought to be facilitated by their tameness and tolerant temperament [5–7]. Dogs are often considered to be a more docile and affectionate, juvenile version of wolves [8–10], and indeed, among human-raised wolves and dogs, the latter seem better at inhibiting their agonistic behaviours and cooperating with humans [11]. Although this increased tolerance in dog–human interactions is probably facilitated by socialization by humans and lifelong experiences of relaxed interactions with them [12], various hypotheses suggest that during domestication dogs have also been selected for reduced aggression and fear in comparison with wolves [5,11,13].

Although this view of dogs having a more tolerant and less aggressive temperament than wolves is based mainly on human–animal interactions, Hare *et al*. [14] have argued that dogs are more tolerant and less aggressive than wolves also when interacting with conspecifics (p. 574; see also [15]). Most other domestication hypotheses remain unclear as to whether they expect the behaviour of dogs to be driven by more tolerant motivations specifically when interacting with humans or whether they see reduced aggressiveness as a general characteristic of dogs that can be expected also in intraspecific contexts. All domestication theories, however, seem to ignore earlier comparisons where apparently contradictory behaviours have been observed in dog and wolf packs. Based on observations of spontaneous social interactions of captive dog and wolf packs raised under identical conditions, dogs of various breeds have been shown to develop more intense aggression than wolves, with serious fights occurring more often in dogs in contrast to the ritualized agonistic behaviours of wolves [9,16–18]. This is so despite the fact that, although in some breeds aggression against strangers has probably been and still is an important basis for selection, wolves appear more aggressive than dogs in intergroup interactions. While feral dog groups rarely engage in physical aggression upon meeting [19,20], and only one single case has been described when an out-group dog was killed after entering the territory of another group [21], aggression of wolves towards non-pack members along the border of their territories can be extreme [22] and is one of the major mortality factors for wolves (after humans) [22,23]. However, within-group aggression and aggression towards out-group members have different functions, and are typically not correlated (see [24] for an intensive discussion).

In this study, we set out to compare tolerant and agonistic behaviours of wolves and dogs towards their pack-mates during cofeeding, a situation that has directly been linked to cooperation in other species. For instance, Hare *et al.* [25] found that dyads of bonobos (*Pan paniscus*) were significantly more likely to cofeed and cooperate than pairs of chimpanzees (*Pan troglodytes*). The difference in tolerance was especially pronounced if the food was placed in a single, monopolizable dish, and while this difference was not reflected in a higher number of aggressive behaviours in chimpanzees compared with bonobos, the authors reported that chimpanzees seemed to avoid each other, whereas bonobos were at ease with the partner (p. 619), which was attributed to a higher mutual tolerance in bonobos. A similar link between cooperation and tolerance has also been reported in macaque species [26]. Less tolerant species, in which dominant animals show more agonistic behaviours towards their subordinate group-mates in a unidirectional manner, appear less cooperative than more tolerant species, characterized with aggression of a lower intensity and a more balanced distribution.

Accordingly, we set out to make a similar comparison of tolerance, a prerequisite for successful cooperation, in dogs and wolves by testing dyads of pack-living dogs and wolves raised and kept under identical conditions, when being fed with either a single bowl of meat pieces or a large bone. Both kinds of food could be shared as well as easily monopolized, although only the bone could be taken away. During the tests, we analysed not only the amount of food monopolization, cofeeding and agonistic behaviours, but especially their distribution over the subordinate and dominant members of the dyads in order to compare the tolerance of dogs and wolves.

## 2. Material and methods

### (a) Subjects

All wolves (*n* = 9) that participated in this study originated from North America but were born in captivity. The dogs (*n* = 8) were mongrels born in animal shelters in Hungary. For sex, age, relatedness and pack assignment refer to table 1. Data were collected from August to December 2009 (wolves) and from April to July 2011 (dogs). In the middle of data collection (on 10 October, when the young animals were five months old) the two packs of wolves were integrated. The dogs lived in stable packs over the entire testing period.

**Table 1.** List of animals, indicating genetic relationships (litters), sex (M, male; F, female), age at testing and pack numbers.

species	animal	sex	litter	age at testing (months)	pack
wolf	Tatonga	F	4	6	1/2^a^
wolf	Nanuk	M	3	6	1/2^a^
wolf	Geronimo	M	5	6	1/2^a^
wolf	Yukon	F	5	6	1/2^a^
wolf	Cherokee	M	6	6	1/2^a^
wolf	Apache	M	6	6	1/2^a^
wolf	Aragorn	M	1	18	2
wolf	Shima	F	1	18	2
wolf dog	Kaspar Rafiki	M M	2 1	18 18	2 3
dog	Maisha	M	2	18	3
dog	Asali	M	3	9	3
dog	Binti	F	3	9	3
dog	Bashira	F	4	9	3
dog	Hakima	M	4	9	3
dog	Kilio	M	2	18	4
dog	Meru	M	5	9	4

^a^After two months of data collection, the young wolves (pack 1) were integrated with three older wolves (pack 2).

All of the animals were hand-raised in a comparable way in peer groups at the Wolf Science Center (WSC) after being separated from their mothers in the first 10 days after birth. All animals were integrated with adult animals into different packs when four to six months old, but contact with humans was maintained in the form of daily training and behavioural test sessions (see [27] for details on the raising procedure).

### (b) Observations

To define the dominance relationships of the animals, we coded submissive and dominant behaviours of the animals from videos of their spontaneous social interactions that were recorded in each pack during the respective testing period. Videos were coded using all occurrence sampling and the WSC ethogram (table 2). Video recordings were randomly distributed over light hours (between 6.00 and 20.00) with at least 2–3 days between them, and were only collected when all members of the packs were present and no disturbance occurred (e.g. pack visits, visitors in the park). A total of 12 videos of pack 1 (5 h 52 min), 18 videos of pack 1 + 2 (after wolf pack formation; 22 h 12 min), 11 videos of pack 3 (5 h 45 min) and 20 videos of pack 4 (3 h 49 min) were analysed.

**Table 2.** Definitions of dominant and submissive behaviours used to define rank relationships.

behaviours	definitions
dominant	
stand tall	to straighten up to full height, with a rigid posture and tail, possibly with raised hackles, ears erect and tail perpendicular or above the back
stand over	to stand over another's body, with all four paws on the ground; receiver may have either the whole body or just the forepaws under the actor's belly/side; tail held high
paw on	to place one or both forepaws on the other's back
ride up (ru)	to mount another one from behind or from the side, exhibiting a thrusting motion
head on (ho)	the subject approaches another's shoulder/back and puts its head on it; most times formation looks like a capital ‘T’
muzzle bite (mz)	to grab the muzzle of another subject either softly or with enough pressure to make the other whimper
submissive	
crouch	to lower the head, sometimes bending the legs, arching the back, lowering the tail between the hind legs and avoiding eye contact
passive submission	to lie on the back showing the stomach and holding the tail between the legs. The ears are held back and close to the head and the subject raises a hind leg for inguinal presentation
active submission	the subject has its tail tucked between the hind legs sometimes wagging it while it is in a crouched position (with hindquarters lowered) and may attempt to paw and lick the side of actors’/aggressor's muzzle; the behaviour may include urination
play submissive	to play with the tail between the hind legs, often running away and snapping at the other
approach submissive	to slowly approach another animal within one body length and remain within that distance for at least 5 s; the approach is characterized by a ducked posture and tail between the legs; subject may move also in a wavy line and in a hesitant (stop–start) manner
withdrawing	to move away slowly from another animal, displaying a submissive posture, having been threatened or attacked, or having had a fight
submissive avoidance	in response to another reducing the distance towards it, the subject moves away displaying a submissive posture; the subject may also look at the individual he is trying to avoid
non-submissive avoidance	in response to being approached by another animal, the subject moves out of its way or changes his direction to move away from the approaching animal
being supplanted	in response to being approached by another animal, the subject leaves the place it has been interested in and moves away

### (c) Experimental set-up

Each animal was tested with each of its pack members in a testing room (3 × 4 m) one to three times in each of the following two conditions:

(1) Meat condition: pieces of raw meat were spread over a large bowl (size was varied according to the size of the animals; wolves: 40 cm diameter; dogs: 20 cm diameter). While the bowls were large enough to allow subjects to eat from the same bowl simultaneously, they were also small enough so that an animal could easily monopolize it. The food could not be carried away.
(2) Bone condition: one single large bone (20–30 cm) was provided. Although it was large enough for more than one animal to chew on it simultaneously, the subjects had the opportunity to carry it away and could easily protect it.

Each trial lasted for a maximum of 5 min. Each animal was tested only once per day. Conditions and combinations of dyads were counterbalanced across and between all individuals within a pack. In order to make sure that we tested only dyads in which the animals had enough time to establish a stable relationship, the wolves were tested only with their original pack-mates also after the two wolf packs were integrated (table 1). All experimental trials were videotaped from outside of the testing room in order to avoid any disturbance of the animals.

### (d) Procedure

To ensure that both animals were at the same distance from the food resource at the beginning of the experimental trial, the food was covered with a square wooden box (45 × 45 cm, 15 cm height) that could be lifted from outside of the room using a string-pulling system. Before the experiment, all animals were habituated to the wooden box, first by placing it into their living enclosure for two weeks and second by letting the animals individually meet the moving box in the experimental room. At this stage, food was hidden under the box, thus the animals could also learn that they could get access to the food once the box was lifted. Once the animals showed no hesitation to approach the box in these individual trials, they proceeded to the testing phase.

Each test trial started with placing the food in the middle of the room and covering it with the wooden box. After this, the two subjects were allowed to enter the room and one experimenter started to record all interactions with a hand-held video camera. Once both animals were standing next to the wooden box (within one body length) with their heads turned towards the box, a second experimenter pulled the box up to the ceiling. The test was terminated when the food was consumed or after 5 min.

### (e) Analyses

The dominance ranks for individuals in each pack were calculated based on the number of their submissive and dominant behaviours (see table 2 for definitions) shown towards other pack members. We used one interaction to establish directionality in any dyad. Individuals were ordered to reduce the number of circular triads [28]. When a relationship between two individuals was unclear, that pair was omitted from the analyses. See the electronic supplementary material for dominance matrices.

Behavioural data collected during the tolerance tests were extracted and analysed using the Observer software (v. 5.0; Noldus Information Technology). From the videotapes, for each individual in each test, we coded the likelihood (0/1) and/or the relative duration of silent cofeeding (i.e. feeding at the same resource without aggressive signals), cofeeding with agonistic signals (i.e. feeding at the same resource while mildly threatening the partner by staring, growling, curling the lips, baring the canines, raising the hackles, snarling, growling and barking, sometimes lifting the tail perpendicularly or above the back), agonistic behaviours (table 3) and feeding alone (i.e. the subject was feeding without the partner at the resource).

**Table 3.** Definitions of agonistic behaviours coded in the tolerance tests.

agonistic behaviours	definitions
threat	to assume a threatening posture: pointing, staring, curling the lips, baring the canines, raising the hackles, snarling, growling and barking, sometimes holding the tail perpendicularly or above the back
charge	to walk towards another wolf with piloerect, stiff forelegs and ears back
attack	to run or jump towards another animal with tail, ears and sometimes hackles up, often biting at the neck or muzzle, forcing it on ground and holding it there
knock-down	to strike another wolf sharply with the chest or shoulder so that the other one falls to the ground
pin	to grab another one at the neck or at the muzzle, forcing it down to the ground and holding it there
fight	a general term for high-intensity, aggressive, often damaging encounters
chase	to run after a conspecific, usually with ears back and piloerect
bite	to move quickly forward and bite by closing the jaws and the teeth on another, possibly accompanied by showing the teeth and eventually growling and barking
snapping	to snap into the air with the flew up so that the teeth are visible

All test videos were analysed by an independent coder. To confirm scoring consistency, 20% of the videos were coded by a second coder. Spearman's rank correlations (*ρ*) were in general high (test duration: 0.99; total number of agonistic interactions: 0.81; duration of cofeeding with agonistic signals: 0.85; duration of non-communicative cofeeding: 0.92; duration of feeding alone: 0.81).

We analysed whether species, test condition (meat or bone), age (in months) and dominance status of the subject (higher- or lower-ranked member of the dyad) influenced the occurrence of tolerant and agonistic cofeeding, feeding alone, and the relative duration of these in the animals that did show the respective behaviours, as well as the relative number of agonistic behaviours. To analyse the occurrence of behaviours, we calculated a generalized linear mixed-effect model (GLMM) using a binomial distribution. The relative duration of the respective behaviours (in the case of silent and agonistic cofeeding only when it occurred) were calculated using linear mixed-effect (LME) models. Since the residuals were not normally distributed, we used square root transformation in the case of agonistic cofeeding, silent cofeeding and feeding alone. In all models, the focal identity and the dyad were included as random effects. The statistical analyses were performed using the program R v. 2.15.2. [29]. All data are provided in the electronic supplementary material.

## 3. Results

### (a) Monopolization of food resource

**Figure 1.**
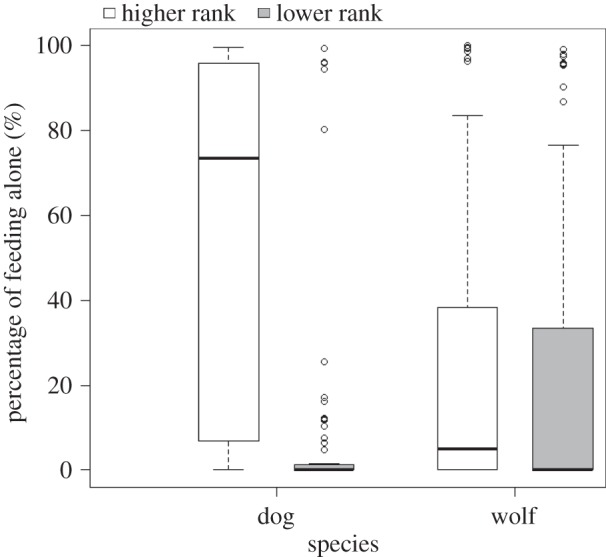
Relative duration (percentage of trial duration; maximum 300 s) of individuals feeding alone (if the behaviour occurred) dependent on their dominance status in the tested dyad (high/low). Boxes represent the interquartile range, bars within boxes are median values and whiskers indicate the 5th and 95th percentiles.

We found an interaction between species and dominance status in the likelihood as well as in the relative duration of feeding alone (likelihood: GLMM: *z* = 2.196, *p* = 0.028; relative duration: LME: *F*_1,74_ = 14.908, *p* < 0.001; figure 1). While higher-ranked dogs were more likely to feed alone and did so longer than lower-ranked dogs (likelihood: GLMM: *z* = −3.854, *p* < 0.001; relative duration: LME: *F*_1,28_ = 39.787, *p* < 0.001), in the wolves we found no influence of rank on the likelihood and duration of feeding alone (likelihood: GLMM: *z* = −1.43, *p* = 0.15; relative duration: LME: *F*_1,47_ = 0.41, *p* = 0.52). In addition, we found an interaction between dominance status and condition in the likelihood and duration of feeding alone (likelihood: GLMM: *z* = 2.218, *p* = 0.027; relative duration: LME: *F*_1,200_ = 9.669, *p* = 0.002). Higher-ranked individuals were more likely to feed alone and did so longer in the bone than in the meat condition (likelihood: GLMM: *z* = −2.793, *p* = 0.005; relative duration: LME: *F*_1,98_ = 41.277, *p* < 0.001). Lower-ranked individuals showed no difference in the likelihood to feed alone; however, they did so for longer in the bone than in the meat condition (likelihood: GLMM: *z* = 0.25, *p* = 0.81; relative duration: LME: *F*_1,98_ = 5.967, *p* = 0.016). Furthermore, we found no influence of age on the occurrence and the duration of feeding alone (likelihood: GLMM: *z* = 3.59, *p* = 0.11; relative duration: LME: *F*_1,61_ = 0.05, *p* = 0.82).

### (b) Agonistic behaviours

**Figure 2.**
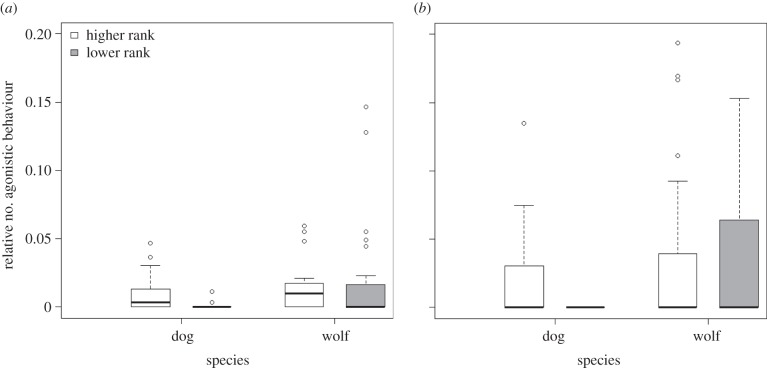
Frequency of agonistic behaviours (1/s) in the two species according to the individuals’ dominance status in the (*a*) bone and (*b*) meat condition. Boxes represent the interquartile range, bars within boxes are median values and whiskers indicate the 5th and 9th percentiles.

Owing to interactions between species and age (GLMM: *z* = 2.860, *p* = 0.004), between species and dominance status (GLMM: *z* = 3.464, *p* = 001), and between species and condition (GLMM: *z* = 2.287, *p* = 0.022), we analysed agonistic interactions in the two species separately. In the dogs, we found no difference between the two conditions (GLMM: *z* = 0.94, *p* = 0.35), and no influence of age (GLMM: *z* = −1.31, *p* = 0.19), but more agonistic behaviours in the higher- than lower-ranked animals (GLMM: *z* = −5.350, *p* < 0.001; figure 2). In the wolves, we found an effect of age on the relative number of agonistic behaviours (GLMM: *z* = 2.723, *p* = 0.006). The younger animals showed more agonistic behaviours than the older ones. Furthermore, we found more agonistic behaviours in the meat than in the bone condition (GLMM: *z* = 5.811, *p* < 0.001), however, we found no influence of dominance status (GLMM: *z* = −0.28, *p* = 0.78). Beyond the distribution of agonistic behaviours, it is worthwhile to note that neither the wolves nor the dogs were very aggressive during testing: agonistic interactions occurred only in 84 of the 260 test sessions (dogs: 36/134; wolves: 48/126). Moreover, of the 92 agonistic behaviours in dogs, 73 were threats, while of the 185 agonistic behaviours observed in wolves, 162 were threats.

### (c) Silent cofeeding

Regarding tolerant behaviours, silent cofeeding was not influenced by status (likelihood: status: GLMM: *z* = −0.15, *p* = 0.88; relative duration: LME: *t*_115_ = −1.22, *p* = 0.22). However, in the likelihood, we found an interaction between species and condition (GLMM: *z* = 2.953, *p* = 0.003). While wolves and dogs behaved similarly in the meat condition (GLMM: *z* = −0.18, *p* = 0.86), dogs were more likely to silently co-feed than wolves in the bone condition (GLMM: *z* = −5.208, *p* < 0.001). In general, silent cofeeding occurred for longer in the meat than in the bone condition (LME: *t*_120_ = 20.717, *p* < 0.001). While age had no influence on the likelihood of silent cofeeding (age: GLMM: *z* = 1.87, *p* = 0.06), we found an interaction between species and age in the duration of silent cofeeding (LME: *t*_13_ = −3.529, *p* = 0.004). While we found no influence of age in dogs (LME: *t*_6_ = 0.63, *p* = 0.55), in wolves the older ones showed longer silent cofeeding than the younger ones (LME: *t*_7_ = −2.995, *p* = 0.020).

### (d) Cofeeding with agonistic signals

We found an interaction between species and status in the likelihood that the behaviour occurred (GLMM: *z* = 2.703, *p* = 0.007), with dominant dogs being more likely to cofeed aggressively than lower-ranking individuals, but with no difference between higher- and lower-ranking wolves (GLMM: dog: *z* = −2.620, *p* = 0.009; wolf: *z* = 0.70, *p* = 0.47). Moreover, while status did not influence the relative duration of cofeeding with agonistic signals in wolves (LME: *F*_1,23_ = 0.14, *p* = 0.71), the behaviour only occurred in higher ranked dogs. Furthermore, the likelihood of agonistic cofeeding was only influenced by condition in the wolves, which cofed more often in the meat than in the bone condition (GLMM: wolf: *z* = 2.531, *p* = 0.011; dog: *z* = 0.37, *p* = 0.71). In general, if cofeeding occurred, agonistic cofeeding lasted longer in wolves than in dogs (LME: *F*_1,38_ = 17.821, *p* < 0.001), and all, wolves and dogs, cofed for longer in the meat than in the bone condition (LME: *F*_1,38_ = 9.176, *p* = 0.004). Finally, we found no influence of age on the occurrence or on the duration of agonistic cofeeding (likelihood: GLMM: *z* = 1.37, *p* = 0.17; relative duration: LME: *F*_1,37_ = 0.18, *p* = 0.67).

## 4. Discussion

To summarize, our results suggest that wolves are more tolerant than dogs because in wolves low- and high-ranking animals monopolized the food and showed agonistic behaviours to a similar extent in contrast to dogs, where food monopolization and threatening the partner were privileges of the high-ranking members of the dyads. In contrast to the subordinate wolves, which readily challenged their dominant partners (e.g. with agonistic signals during cofeeding), the low-ranking dogs cofed only silently and readily retreated when rebuked by the dominant partner. In sum, in our captive packs, wolves behaved tolerantly to their pack members during feeding, in contrast to the dogs, which have a steeper and more rigid dominance hierarchy. At the same time, however, dogs and wolves proved to be similar in their agonistic behaviour, displaying mostly threatening signals and even those in only one third of the tests.

As mentioned briefly in the introduction, similar differences in the distribution of agonistic behaviours over low- and high-ranking animals have also been described between other closely related species. More specifically, based on their agonistic behaviour, tolerance, conciliatory behaviour, dominance gradient and kin bias, Thierry [26] arranged macaque species according to a four-grade scale. The first grade is characterized by unidirectional aggression of dominant animals, with high and severe biting rates, and subordinates generally fleeing or submitting when attacked. The species belonging here are characterized as having a steep dominance hierarchy and a low tolerance level. At the other extreme of the scale, the intensity of aggression and the biting rate are low, and most agonistic interactions are bidirectional, meaning that the victim of aggression protests or counter-attacks. In these species, the dominance gradient is less steep and tolerance is high. Thus, while the asymmetry of contests and the dominance gradient decrease from the first to the fourth grade, social tolerance increases. In accordance with Thierry's [26] categorization, based on our results dogs would be characterized as less tolerant than wolves. As we tested a relatively low number of animals living in few packs at the same facility, one may question to what extent these findings are representative for wolves and dogs in general. Importantly, earlier observations on human-raised wolves and dogs by Frank & Frank [9] and Feddersen-Petersen [17,30] also reported more fierce intraspecific aggression in young dogs than in young wolves.

One may argue, however, that our results reflect rather the young age of our subjects than a more fundamental difference in the tolerance of dogs and wolves. Domestication is thought to accelerate sexual maturation [31], and accordingly, wolves are usually considered to reach sexual maturity later than dogs. One might argue that higher tolerance in our wolves may reflect their lack of maturation. Testing older animals would indeed be important, and one may expect them to be more aggressive. However, our findings, although on a small sample (we had only three wolves in the older age group), contradict this expectation: older wolves were more likely to feed together silently than the younger wolves, suggesting that tolerance actually increased with age rather than decreased.

Therefore, the question remains why dogs behave less tolerantly towards their conspecific pack-mates than wolves. First, the steeper dominance hierarchy of dogs may result from their higher intraspecific aggressiveness compared with wolves, as suggested by earlier observations [9,17,30]. More frequent or more intensive aggression in dog packs than wolf packs may reflect a less tolerant temperament of dogs than of wolves, which would be in sharp contrast with suggestions of the domestication hypotheses [11,14]. Alternatively or additionally, however, dogs may also be more sensitive than wolves to agonistic behaviours of their conspecifics. In this case, even if dogs and wolves do not differ in the frequency and intensity of aggression, dogs can be intimidated more easily than wolves, and consequently will be likely to learn to avoid potential conflicts during their development. If so, the less tolerant behaviour of dogs compared with wolves could reflect a more sensitive temperament rather than a less tolerant one. Further studies, comparing the early agonistic interactions of dog and wolf pups, are needed to clarify if either or both of these two explanations are correct.

Before making any conclusions about fundamental differences in the social temperament of dogs and wolves, it is important to realize that other differences between dogs and wolves may also explain their differently tolerant behaviour.

First, domestic dogs may handle competitive situations around resources on a case-by-case basis by using violence to establish control rather than by relying on the dominance relationships of the interacting partners. This is unlikely, however, given that in free-ranging dogs dominance relationships remain stable across different competitive contexts, and access to food resources is predicted reasonably well by the rank positions of the individuals, with high-ranking individuals having priority of access [32] (see also [33,34]). Stable dominance hierarchies have also been reported for groups of pet dogs [35,36]. Moreover, according to this hypothesis, the agonistic behaviours shown by each of our dogs should be independent of their social rank. By contrast, our results showed that the dominant dogs showed more agonistic behaviours than the subordinates. One can, however, still argue that instead of a functional relationship between the two, dominance rank and showing agonistic behaviours to a partner simply correlate across individuals in dogs.

Second, Feddersen-Petersen [30] suggested that visual communication in dogs is somewhat impaired due to their reduced visual (facial as well as bodily) expression caused by their altered morphology (fur colouring and length, head shape, hanging ears, lack of tail, etc.; see also [37]). As a consequence, this impairment might lead to an inability to control conflicts at close quarters, which might appear to the observer as if the dogs had a higher motivation to initiate and escalate conflicts, while in truth they just have no means to communicate properly with each other, and thus to de-escalate conflicts. In this study, we used the same ethogram to code the behaviour of the dogs and of the wolves. While dogs showed all behaviours except knock-down, bite and snapping, wolves did not ‘pin’ or ‘fight’ (for definitions see table 3). Nevertheless, although dogs and wolves seem to use the same signals overall, it is possible that dogs do not use them as appropriately as wolves.

Whichever mechanistic explanation (less tolerant or more sensitive temperament, impaired signalling, or non-functional dominance hierarchy) is true, our and former observations that domestic dogs show a less tolerant behaviour towards their group-mates and express a steeper dominance hierarchy than wolves in a feeding context nicely fit the social ecology of wolves and dogs. While free-ranging domestic dogs have retained some similar behavioural patterns (e.g. living in pack-like groups and forming stable hierarchical structures [32,34]), they differ from wolves in several aspects. For example, they are not organized as family units but rather as multi-male/multi-female groups of largely unrelated individuals. Accordingly, female dogs usually raise their offspring alone or with limited help from the father [38]. Moreover, dogs also differ from wolves in their foraging strategies, with wolves relying heavily on hunting, while dogs often feed on stable food resources provided by humans (e.g. scavenging at rubbish dumps or food provisioned by humans [39,40]; but see [41]). It has been suggested that, in dogs, this feeding ecology might have relaxed the need to feed quickly, whereas wolves need to gorge food down to avoid competitors (bears, ravens) taking away their food, and thus cannot engage in conflicts over food. Alternatively, this buffering effect of food provisioning by humans has been proposed to reduce selection against intraspecific aggression in dogs [9], which in turn might explain their difficulties in cooperating with each other and resolving social conflicts [17,30]. Interestingly, from 6 to 12 months of age, dogs seem to be similarly aggressive to jackals adapted to a more solitary life [17].

In contrast to the social system of free-ranging dogs, the social life of wolves is characterized by the cooperation of closely related animals. The wolf pack is a family unit, where the offspring from the previous year delay dispersal and help the breeding pair(s) to raise their young [42,43]. Moreover, they rely on close action coordination with pack members when defending their kills [44] and territories, and hunting large game [42,43]. Accordingly, selection has probably favoured reduced aggression (by high tolerance, fine-tuned communication and a functional dominance hierarchy) towards close kin, allowing them to cooperate closely with each other.

Consequently, if we relate our experimental findings on the tolerance of wolves to the social ecology of wild-living packs, we find the same link between tolerance and intraspecific cooperation as in other species (e.g. [26,45]): wolves appear tolerant, attentive and at the same time cooperative towards their pack members [42,46]. This view is also supported by our recent results showing that wolves follow the gaze of conspecifics [47] and are more adept at socially learning from conspecifics than dogs [27]. This view of wolves is in contrast to the starting point of several recent domestication hypotheses describing wolves as less cooperative than dogs [11,14]. Instead, we propose that wolves possess most of the skills that have been suggested to be preconditions for successful cooperation. Therefore, dog–human cooperation might have evolved on the foundation of wolf–wolf cooperation (‘canine cooperation hypothesis’ [27,48,49]). A first step might have been that dogs lost their fear of humans and thus became able to extend their relevant social skills to interactions with them (see also [5]).

The social life of wild-living dog packs (as well as our and former findings on the intraspecific social behaviour of captive dog packs) show, however, that this could not all have been as indicated by the less tolerant behaviour of dogs towards conspecifics in comparison to wolves. Further research has to clarify (i) what explains this behavioural difference at a mechanistic level, as well as (ii) to what extent it reflects different genetic predispositions in dogs and wolves, which are then differently enlarged by developmental processes (e.g. socialization in differently tolerant social groups). As discussed earlier, socialization in differently tolerant conspecific groups can strongly influence the behaviour of adult animals, and the behaviour of dogs that grow up in human families can be even more strongly modified. Moreover, it is important to note that the canine cooperation hypothesis is compatible also with other evolutionary hypotheses that specifically address the human-directed behaviour of dogs.

Still, we argue that studying the intraspecific social life of the domestic dog can provide important information about the effects of domestication by differentiating between general characteristics of dogs and their other skills used only when interacting with humans. Even more, we suggest that such studies can give us a more complete insight into the social ecology of dogs, which has probably driven the evolution of their social behaviour and the cognitive and emotional processes underlying it. Living together with (or close to) humans, cooperating and communicating with them has certainly imposed important adaptational demands on the evolution of dog behaviour [50,51]. Beyond this, however, living in conspecific groups and interacting with other dogs were always part of the life of domestic dogs: pet dogs represent a small part of the entire dog population, with current estimates suggesting that free-ranging dogs represent about 76–83% of the global dog population [52,53]. These millions of dogs live more or less independently from humans, in conspecific groups in which their survival is greatly determined by successful communication and social manoeuvring in intraspecific contexts [54].

## Supplementary Material

Supplementary data

## Supplementary Material

Raw Data
