# Supplementary material for: Testing the myth: tolerant dogs and aggressive wolves
Source: Proc Biol Sci. 2015 May 22;282(1807):20150220. doi: 10.1098/rspb.2015.0220 (PMC4424647; doi:10.1098/rspb.2015.0220)
Supplement: Supplementary data [file rspb20150220supp1.doc]

**Supplemental Material**

**Dominance Matrices**

The dominance ranks for individuals in each pack were calculated based on the number of their submissive and dominant behaviours (see table 2 for definitions) shown to other pack members. We used one interaction to establish directionality in any dyad. Individuals were ordered to reduce the number of circular triads (de Vries 1995). When a relationship between two individuals was unclear, that pair was omitted from the analyses.

WOLVES:

**Pack 1 (used for tolerance tests conducted from 31.7. – 9.10. 2009)**

(underlined numbers reflect inconsistencies)

🡪 increasing dominance rank

|  | **apa** | **che** | **tat** | **yuk** | **ger** | **nan** |
| --- | --- | --- | --- | --- | --- | --- |
| **apa** |  | 6 | 5 | 7 | ***2*** | 8 |
| **che** | 4 |  | 14 | 3 | 4 | 19 |
| **tat** | 4 | 10 |  | 10 | 9 | 16 |
| **yuk** | 7 | 0 | 5 |  | 4 | 10 |
| **ger** | ***3*** | 0 | 1 | 0 |  | 38 |
| **Nanuk** | 0 | 0 | 0 | 0 | 1 |  |
|  |  |  |  |  |  |  |

Excluded from analyses Yukon/Apache due to unresolved relationship.

**Pack 1 + 2 (after pack formation; used for tolerance tests conducted between the 10.10. – 26.11.2009)**

🡪 increasing dominance rank

|  | **tat** | **apa** | **che** | **yuk** | **ger** | **nan** | **shi** | **ara** | **kas** |
| --- | --- | --- | --- | --- | --- | --- | --- | --- | --- |
| **tat** |  | 3 | 2 | 2 | 9 | 3 | 37 | 91 | 26 |
| **apa** | 0 |  | 3 | 5 | 8 | 19 | 40 | 32 | 49 |
| **che** | 0 | 2 |  | 4 | 9 | 14 | 6 | 26 | 41 |
| **yuk** | 0 | 1 | 0 |  | 5 | 2 | 23 | 52 | 40 |
| **ger** | 0 | 1 | 1 | 0 |  | 23 | 10 | 24 | 14 |
| **nan** | 0 | 1 | 0 | 0 | 7 |  | 7 | 10 | 3 |
| **shi** | 0 | 0 | 0 | 0 | 2 | 0 |  | 15 | 6 |
| **ara** | 0 | 0 | 0 | 0 | 1 | 0 | 0 |  | 11 |
| **kas** | 0 | 0 | 0 | 0 | 5 | 0 | 0 | 0 |  |

DOGS

Pack 3 (used for all tolerance tests conducted with animals from pack 3)

🡪 increasing dominance rank

|  | **Hakima** | **Bashira** | **Binti** | **Asali** | **Maisha** | **Rafiki** |
| --- | --- | --- | --- | --- | --- | --- |
| **Hakima** |  | 9 | 3 | 6 | 14 | 58 |
| **Bashira** | 2 |  | 2 | 7 | 6 | 57 |
| **Binti** | 0 | 2 |  | 4 | 3 | 37 |
| **Asali** | 0 | 0 | 0 |  | 15 | 31 |
| **Maisha** | 0 | 1 | 1 | 0 |  | 54 |
| **Rafiki** | 0 | 0 | 0 | 0 | 0 |  |

|  |  |  |  |  |  |  |
| --- | --- | --- | --- | --- | --- | --- |

Excluded from analyses Bashira/Binti due to unresolved relationship.

Pack 4: (used for all tolerance tests conducted with animals from pack 4)

🡪 increasing dominance rank

|  | Meru | Kilio |
| --- | --- | --- |
| Meru | 0 | 40 |
| Kilio | 1 | 0 |

**References**
